# Time Since Stroke Onset, Quantitative Collateral Score, and Functional Outcome After Endovascular Treatment for Acute Ischemic Stroke

**DOI:** 10.1212/WNL.0000000000200968

**Published:** 2022-10-11

**Authors:** Simone M. Uniken Venema, Lennard Wolff, Sophie A. van den Berg, Hendrik Reinink, Sven P.R. Luijten, Hester F. Lingsma, Henk A. Marquering, Anna M.M. Boers, Joost Bot, Sebastiaan Hammer, Paul J. Nederkoorn, Yvo B.W.E.M. Roos, Charles B.L.M. Majoie, Jan Willem Dankbaar, Aad van der Lugt, H. Bart van der Worp, on behalf of MR CLEAN Registry Investigators

**Affiliations:** From the Departments of Neurology and Neurosurgery (S.M.U.V., H.R., H.B.v.d.W.), Brain Center, and Department of Radiology (J.W.D.), University Medical Center Utrecht; Departments of Radiology & Nuclear Medicine (L.W., S.P.R.L., A.v.d.L.), Public Health (H.F.L.), Erasmus MC, University Medical Center, Rotterdam; Departments of Neurology (S.A.v.d.B., P.J.N., Y.B.W.E.M.R.), Radiology and Nuclear Medicine (H.A.M., A.B.), Radiology and Nuclear Medicine (J.B., C.B.L.M.M.), and Biomedical Engineering & Physics (H.A.M., A.M.M.B.), Amsterdam University Medical Centers, University of Amsterdam; and Department of Radiology (S.H.), Haga Ziekenhuis, The Hague, the Netherlands

## Abstract

**Background and Objectives:**

In patients with ischemic stroke undergoing endovascular treatment (EVT), time to treatment and collateral status are important prognostic factors and may be correlated. We aimed to assess the relation between time to CT angiography (CTA) and a quantitatively determined collateral score and to assess whether the collateral score modified the relation between time to recanalization and functional outcome.

**Methods:**

We analyzed data from patients with acute ischemic stroke included in the Multicenter Randomized Controlled Trial of Endovascular Treatment for Acute Ischemic Stroke Registry between 2014 and 2017, who had a carotid terminus or M1 occlusion and were treated with EVT within 6.5 hours of symptom onset. A quantitative collateral score (qCS) was determined from baseline CTA using a validated automated image analysis algorithm. We also determined a 4-point visual collateral score (vCS). Multivariable regression models were used to assess the relations between time to imaging and the qCS and between the time to recanalization and functional outcome (90-day modified Rankin Scale score). An interaction term (time to recanalization × qCS) was entered in the latter model to test whether the qCS modifies this relation. Sensitivity analyses were performed using the vCS.

**Results:**

We analyzed 1,813 patients. The median time from symptom onset to CTA was 91 minutes (interquartile range [IQR] 65–150 minutes), and the median qCS was 49% (IQR 25%–78%). Longer time to CTA was not associated with the log-transformed qCS (adjusted β per 30 minutes, 0.002, 95% CI −0.006 to 0.011). Both a higher qCS (adjusted common odds ratio [acOR] per 10% increase: 1.06, 95% CI 1.03–1.09) and shorter time to recanalization (acOR per 30 minutes: 1.17, 95% CI 1.13–1.22) were independently associated with a shift toward better functional outcome. The qCS did not modify the relation between time to recanalization and functional outcome (*p* for interaction: 0.28). Results from sensitivity analyses using the vCS were similar.

**Discussion:**

In the first 6.5 hours of ischemic stroke caused by carotid terminus or M1 occlusion, the collateral status is unaffected by time to imaging, and the benefit of a shorter time to recanalization is independent of baseline collateral status.

In patients with acute ischemic stroke caused by a proximal occlusion of an intracranial artery, collateral blood flow is essential to sustain the viability of hypoperfused but potentially salvageable tissue distal to the occluded artery.^1^ Hence, the collateral status before endovascular treatment (EVT) is an important determinant of functional outcome, and patients with good collaterals on baseline neuroimaging may also derive greater benefit from this treatment.^2,3^

In the first hours after stroke onset, there is substantial interindividual variability in collateral status on neuroimaging,^4^ which may be explained in part by time from stroke onset to imaging. A longer time from stroke onset has been associated with better^5,6^ and with poorer collaterals,^7^ suggesting either improvement or deterioration of the collateral status over time.

Besides collateral status, time to recanalization is a strong prognostic determinant for functional outcome after EVT, since the proportion of salvageable tissue declines over time,^8^ a process that is likely dependent on the collateral status.^9,10^ Patients with a poorer collateral status require more rapid recanalization to achieve a good functional outcome compared with patients with good collateral status.^11‐13^ However, it is unclear whether collateral status modifies the association between time to recanalization and functional outcome after EVT.^11,13,14^

Collateral status is usually determined using crude visual grading scales with moderate interobserver agreement and variable cross-correlation among different grading methods.^15–17^ Automated quantification of the collateral capacity with artificial intelligence-based image analysis algorithms can be used to score collaterals in a more precise and rater-independent manner.^18^ We determined a quantitative collateral score (qCS) in patients included in the Multicenter Randomized Controlled Trial of Endovascular Treatment for Acute Ischemic Stroke (MR CLEAN) Registry with the aim to investigate the relation between time to baseline neuroimaging and collateral status and to assess whether collateral status modifies the relation between time from symptom onset to recanalization and functional outcome.

## Methods

### Study Population

**Figure 1:**
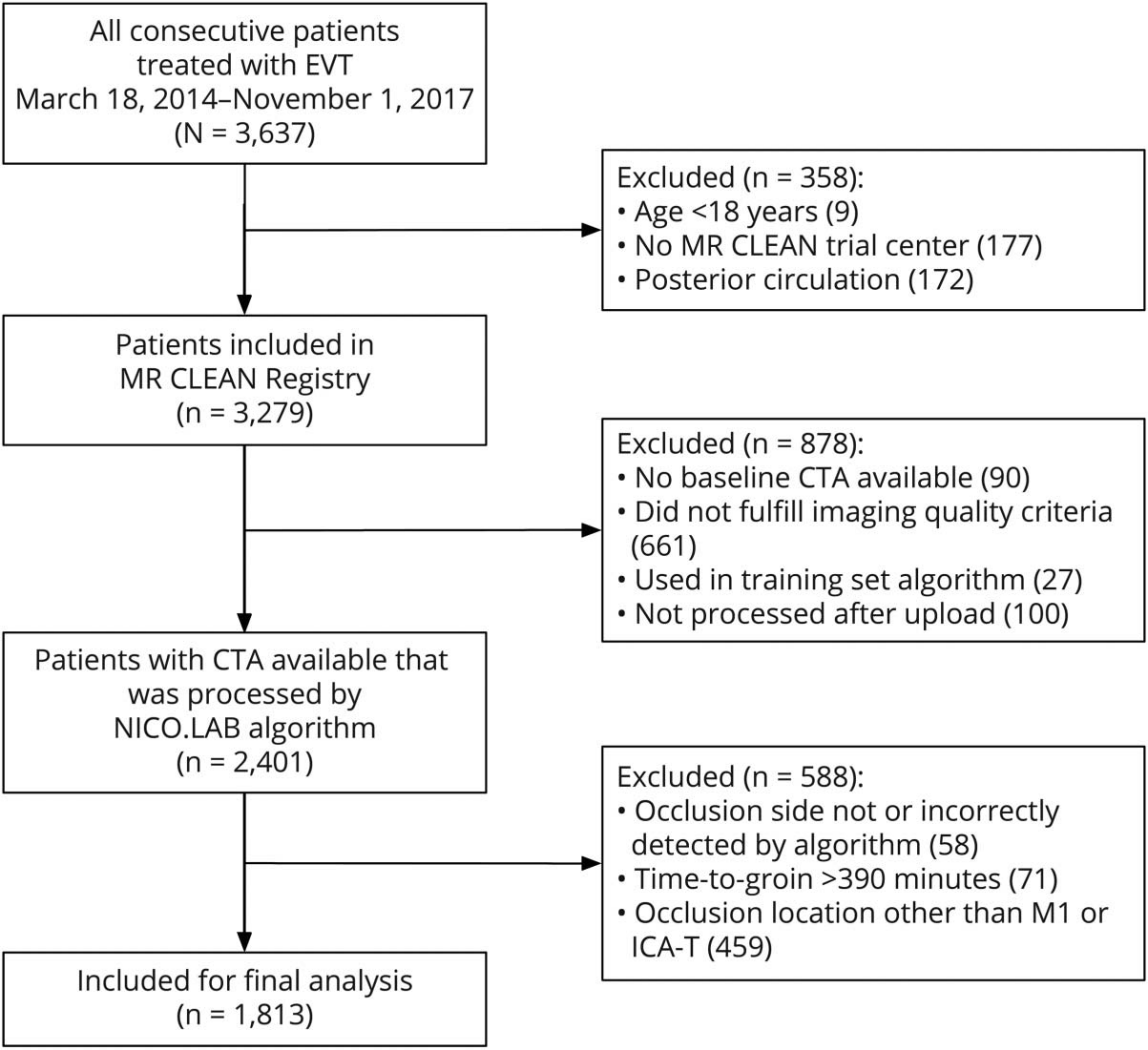
Flowchart of Patients Through the Selection Process CTA = CT angiography; DSA = digital subtraction angiography; EVT = endovascular treatment; ICA(-T) = internal carotid artery (-terminus); MR CLEAN = Multicenter Randomized Controlled Trial of Endovascular Treatment for Acute Ischemic Stroke.

The MR CLEAN Registry is a prospective, observational, multicenter registry of patients with acute ischemic stroke treated with EVT in the Netherlands.^19^ The present study is based on data from patients included in the MR CLEAN Registry between March 16, 2014, and November 1, 2017. Patients were included in the MR CLEAN Registry if they fulfilled the following inclusion criteria: age 18 years or older; diagnosis of acute ischemic stroke with a large vessel occlusion in the anterior circulation confirmed by CT angiography (CTA), magnetic resonance angiography or digital subtraction angiography; and treatment with EVT in an MR CLEAN intervention center. Patients were excluded from the present study if the time from symptom onset to groin puncture exceeded 390 minutes; if the occlusion location was any other than carotid artery terminus (ICA-T) or the M1 segment of the middle cerebral artery (for the purpose of obtaining a uniform sample); if no qCS could be obtained due to insufficient image quality or if no baseline CTA was available for the assessment of the qCS; if the CTA was included in the training sets of the image analysis algorithm; or if the software did not correctly identify the affected hemisphere (Figure 1). If the side of the occlusion, as identified by the software, was deviant from that as identified by the imaging core laboratory, the CTA was again assessed, and the true occlusion location was determined by consensus.

### Standard Protocol Approvals, Registrations, and Patient Consents

The study protocol was evaluated by the Erasmus Medical Center ethics committee in Rotterdam, the Netherlands, which granted permission to conduct the study as a registry (MEC-2014-235). The requirement for written informed consent was waived, but patients were provided with information on the study and were given the opportunity to refuse participation.

### Assessment of the Collateral Circulation

#### Quantitative Collateral Assessment

Collateral circulation was quantitatively assessed on baseline CTA in an automated fashion using StrokeViewer software (version 2.1.22).^20^ The algorithm is based on a previously developed quantitative method for collateral grading in patients with a large vessel occlusion.^21^ The software first locates the ischemic hemisphere using an occlusion detection algorithm^22^ and segments the vessels (arteries) distal to it. This is achieved by convolutional neural networks designed and trained specifically for these tasks. In each hemisphere, the total volume of vasculature segmented by the algorithm (vascular appearance) was determined. The qCS, in turn, was calculated as the ratio of vascular appearance of the ipsilateral hemisphere (distal to the occlusion, within the affected middle cerebral artery territory) to the contralateral hemisphere (eFigure 1, links.lww.com/WNL/C223). The acquisition phase was determined in an automated fashion based on a comparison of the Hounsfield units (HUs) within 2 specific regions of interest in the unaffected hemisphere (namely, the internal carotid artery and the transverse sinus) and categorized as early arterial, late arterial, equilibrium, early venous, or late venous.^14,23^ If the density was higher in the carotid artery than in the transverse sinus (and transverse sinus ≤200 HU), the phase was early arterial. If the density was higher in the transverse sinus in comparison to the carotid artery (and carotid artery ≤200 HU), the phase was late venous. The software has been shown to perform similar to expert radiologists in determining a good vs poor collateral status and is also similar in predicting functional independence at 90 days in patients with a large vessel occlusion.^24^

#### Visual Collateral Assessment

An imaging core laboratory, consisting of experienced (interventional) neuroradiologists, blinded to all clinical findings except the symptom side, determined the collateral score on baseline CTA with a previously used scoring method.^25^ This is a 4-point visual grading scale, with 0 for absent collaterals (0% filling of the vascular territory downstream of the occlusion), 1 for poor collaterals (>0% and ≤50% filling), 2 for moderate collaterals (>50% and <100% filling), and 3 for excellent collaterals (100% filling). Interobserver agreement for this method has previously been determined and was found to be moderate (k = 0.60).^2^

### Clinical Data and Time Variables

Clinical data at baseline and during hospital stay were collected by study personnel at each participating center. The modified Rankin Scale (mRS) score^26^ at 90 days poststroke, as a measure of functional outcome, was assessed by trained local study personnel through a telephone interview with patients or their representatives. This score ranges from 0 (no symptoms) to 6 (death), with a higher score indicating greater disability.

Time to CTA was defined as the time between the onset of symptoms or last seen well and acquisition of the CTA. Time to groin puncture was defined as the time between onset of symptoms or last seen well and arterial puncture in the angiography suite. Time to recanalization was calculated as the time elapsed between onset of symptoms or last seen well and successful recanalization at the end of EVT, or, in case on unsuccessful recanalization, the last contrast bolus. Recanalization was considered successful if the extended Thrombolysis in Cerebral Infarction (eTICI) score^27^ at the end of EVT was 2B, 2C, or 3.

### Statistical Analysis

Baseline characteristics of the study population were compared according to their baseline qCS, categorized as ≤25%, 26%–50%, 51%–75%, and ≥76%, using descriptive statistics. In all multivariable models, adjustments were made for potential confounders selected with a backward elimination approach: starting with a full model, the least significant variables were sequentially removed, until only variables significantly associated with the outcome variable (*p* < 0.05) remained in the model. Variables used in the multivariable models were >98% complete, except baseline glucose (88% complete) and whether general anesthesia was used during the procedure (94% complete). Under the assumption of missing at random, missing values were replaced with imputed values after performing multiple imputations (n = 5) based on relevant variables.

The relation between time to CTA and the qCS was assessed in a multivariable linear regression model with qCS as the outcome variable. The qCS was log10 transformed to approximate a normal distribution of the residuals, after adding 1 point to all qCSs, so that the log10-transformed qCS of 0 would remain 0.^28^ Adjusted β-coefficients and 95% CIs, indicating the percentage change in the qCS, were estimated for each 30-minute increase in time to CTA.

The relations between the qCS and functional outcome and between time to recanalization and functional outcome were determined with ordinal logistic regression using the full mRS score as the outcome variable. To test whether the relation between time to recanalization and functional outcome was modified by collateral status, an interaction term (qCS × time to recanalization) was added to the model, and the likelihood ratio test was used to assess whether the model containing the interaction term was significantly better than the model without. Adjusted common odds ratios (acORs) and 95% CI were estimated for each 10% increase in the qCS and for each 30-minute increase in time to recanalization.

The following sensitivity analyses were performed:

1. All analyses were repeated using the visual collateral score (vCS) instead of the qCS.
2. All analyses were repeated after excluding patients with a suboptimal CTA acquisition phase (early arterial or late venous) or a CTA slice thickness >2 mm.
3. For relation between time to CTA and the qCS: analyses were repeated after excluding patients without a witnessed stroke onset (i.e., where time of last seen well was used to calculate time from stroke onset to CTA).
4. For the relations between the qCS and time to recanalization with functional outcome: analyses were repeated after excluding patients in whom no successful recanalization was achieved after EVT (eTICI ≤2A).

Statistical analyses were performed in R software (version 3.6.1. R Foundation).

### Data Availability

Syntax and output from statistical analyses can be made available on reasonable request to the corresponding author, but individual patient data cannot be distributed.

## Results

### Baseline Characteristics

Of 3,279 patients included in the MR CLEAN Registry, 2,401 had a suitable baseline CTA available that could be processed by the automated algorithm. A total of 1,813 patients were included in the final analysis, after applying our study-specific exclusion criteria (Figure 1), and were similar compared with patients excluded for reasons related to imaging (eTable 1, links.lww.com/WNL/C223). The median qCS was 49% (interquartile range [IQR] 25%–78%). The median time from onset to CTA was 91 minutes (IQR 65–150 minutes), and the median onset to recanalization time was 249 minutes (IQR 198–310 minutes). Patients with a higher baseline qCS were younger and more often female (Table 1). Moreover, patients with a higher qCS had a lower NIH Stroke Scale (NIHSS) score and a higher Alberta Stroke Program Early CT Score (ASPECTS) at baseline, and they less often had an occluded ICA-T segment, as opposed to M1.

**Table 1:**
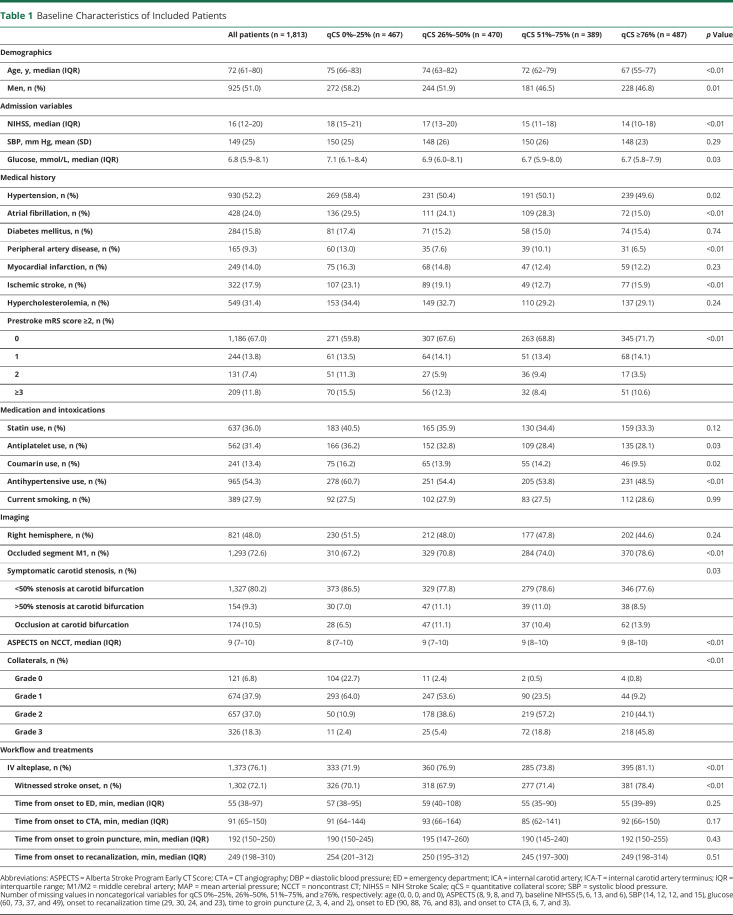
Baseline Characteristics of Included Patients

### Relation Between Time to CTA and Collateral Score

In univariable analysis, there was no association between time to CTA and the log-transformed qCS (β per 30 minutes, 0.001 [−0.007 to 0.010]), and this did not change after adjusting for confounding variables (adjusted β per 30 minutes, 0.002 [−0.006 to 0.011]): for each 30-minute increase in time to CTA, the qCS increases with 0.005% (95% CI decrease of 0.014%–increase of 0.025%). Multivariable analyses were adjusted for age, sex, glucose, occlusion location, previous stroke, smoking, admission diastolic blood pressure, and carotid artery disease.

These results were similar for the vCS (eTable 2, links.lww.com/WNL/C223). Results were also similar after excluding patients without a witnessed stroke onset and after excluding patients with a suboptimal CTA acquisition phase (early arterial or late venous) or a CTA slice thickness >2 mm (eTable 2).

### Relation Between Collateral Score and Time to Recanalization With Functional Outcome

**Figure 2:**
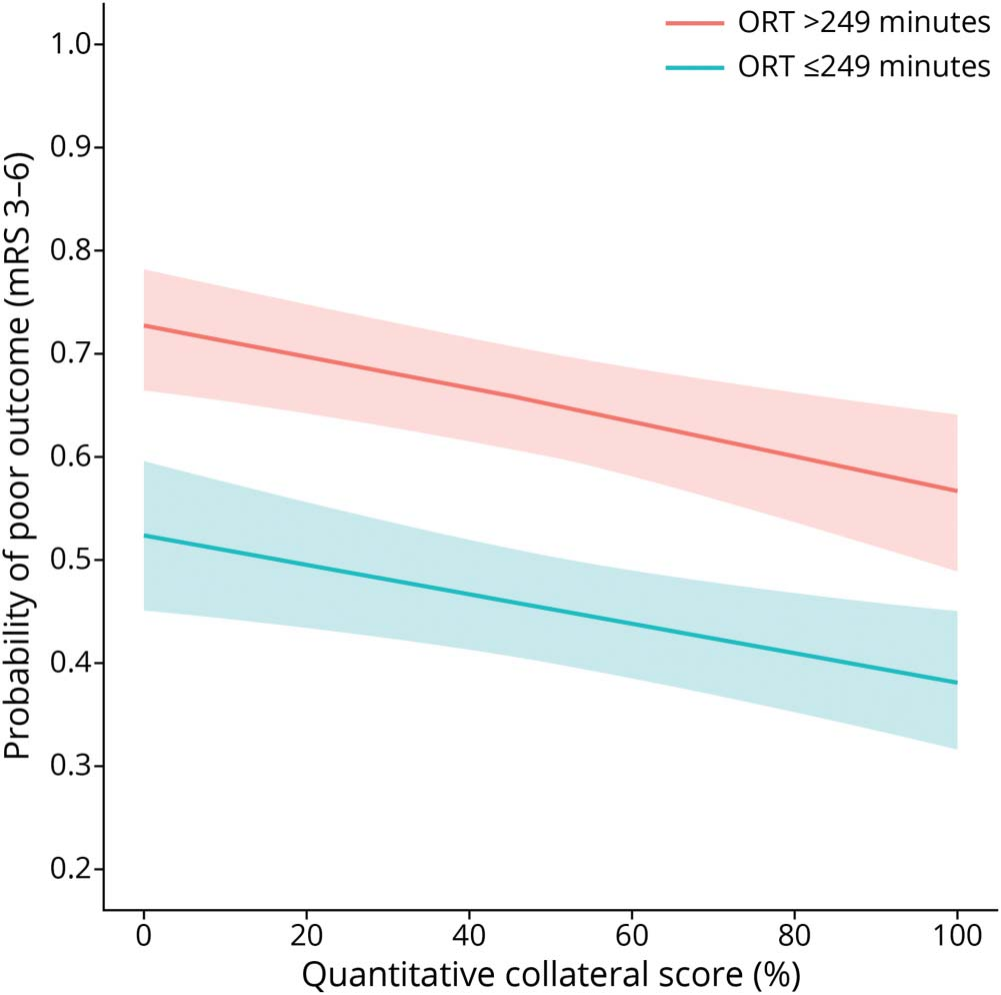
Predicted Probability of Poor Functional Outcome According to the Quantitative Collateral Score and Onset to Recanalization Time Figure is based on a multivariable ordinal logistic regression with the modified Rankin Scale score as an outcome variable. The model was adjusted for potential confounders, and an interaction term was added (ORT ≤249 minutes vs >249 minutes × qCS). ORT was dichotomized at the median for the entire population (249 minutes) for the purpose of this figure only. mRS = modified Rankin Scale score; ORT = onset to recanalization time; qCS = quantitative collateral score.

An increase in the qCS was associated with a shift toward better functional outcome in univariable (acOR per 10% increase in the qCS, 1.15 [1.12–1.18]) and multivariable analysis (acOR per 10% increase in the qCS, 1.06 [1.03–1.09]). An increase in time to recanalization was associated with a shift toward poor functional outcome in univariable analysis (acOR per 30 minutes, 1.17 [1.14–1.21]) and multivariable analysis (acOR per 30 minutes, 1.17 [1.13–1.22]). Multivariable analyses were adjusted for age, glucose, occlusion location, diabetes mellitus, smoking, statin use, admission systolic blood pressure, premorbid modified Rankin Scale score, carotid artery disease, IV thrombolysis, ASPECTS, and baseline NIHSS. The qCS did not modify the association between time to recanalization and functional outcome (*p* for interaction = 0.48; Figure 2 and eFigure 1, links.lww.com/WNL/C223).

These results were similar for the vCS (eTable 3, links.lww.com/WNL/C223). Results were also similar after excluding patients without successful recanalization and after excluding patients with a suboptimal CTA acquisition phase (early arterial or late venous) or a CTA slice thickness >2 mm (eTable 3).

## Discussion

In this prospective cohort study of 1,813 patients with acute ischemic stroke due to large vessel occlusion in the anterior circulation treated with EVT, we did not find a relation between time to imaging and the qCS, suggesting that collateral blood flow remains stable in the first 6 hours after stroke onset. Furthermore, the qCS did not modify the relation between time to recanalization and functional outcome, indicating that a shorter onset to recanalization time is equally beneficial regardless of the qCS. In line with previous studies, better collateral status^3,29^ and a shorter time to recanalization^3,8^ were independent predictors of a better functional outcome.

A few previous studies have assessed the relation between time to imaging and collateral status. In a study of 355 patients with a proximal occlusion in the anterior circulation assessed within 6 hours of stroke onset, longer times from symptom onset were associated with a better collateral status, supporting the hypothesis that additional collaterals may be recruited in a time-dependent manner.^5^ A similar trend toward better collateral status with longer times after stroke onset was found in another study of 134 patients with a proximal middle cerebral artery occlusion.^6^ Others have, however, reported poorer collateral status with longer delays to imaging, suggesting diminishing of collateral flow over time,^7^ or no relation between collateral status and time from symptom onset.^30^ These previous studies were smaller than ours, and collateral status was graded using traditional visual grading scales, as opposed to our automated method.

Based on our findings, we speculate that the observed variability in collateral status after a proximal arterial occlusion in the first 6 hours after symptom onset is individually determined and not related to the time of imaging. Collateral status has been reported to vary depending on, among others, genetic factors^31^ and age.^32^ It is also possible that more time is needed for collaterals to adapt through remodeling.^33^ In our study, with a median time from symptom onset to CTA of 82 minutes, we mainly assessed the presence of preexistent, native collaterals.^33^ We cannot exclude the possibility that the effect of time on collateral status may only become evident beyond the first 6.5 hours. Overall, studies examining collaterals at multiple time points are scarce, and the evolvement of collateral flow over time and the effect of vessel recanalization on this process remain to be studied in more detail.

The absence of an interaction between the qCS and time to recanalization in relation to functional outcome indicates that the detrimental effect of longer time to recanalization is equal across the range of the qCS. This conflicts with several previous studies, which concluded that the association between time to recanalization and functional outcome differed according to baseline collateral status, such that the likelihood of good outcome dropped faster with increasing time in those with a poor collateral status.^11–13^ These studies dichotomized collateral status into good vs poor,^10–12^ only included patients with successful recanalization,^10,11^ or were performed before EVT was implemented in routine clinical practice.^10,11^ Furthermore, these studies merely compared the association of onset to recanalization time with outcome in subgroups of patients with poor or good collateral status but did not assess a statistical interaction between collateral status and time to recanalization. A previous study based on the MR CLEAN Registry, with less strict eligibility criteria and examining the vCS only, concluded that the relation between time to treatment and functional outcome was independent of collateral status, similar to our study.^14^ Nonetheless, patients with both a low qCS and longer time to recanalization had the poorest outcomes in our cohort. In other words, the time window for having a reasonable probability of achieving a good functional outcome is shorter for patients with a poor collateral status.

The present study has several strengths and limitations. Strengths include the large sample size and detailed data set. The consecutive inclusion of patients treated in routine clinical practice ensured that our cohort consists of an unselected group of EVT-eligible patients, which enhances generalizability and limits selection bias. We used 2 distinct methods for the assessment of collateral status, one in an automated fashion using artificial intelligence-based algorithms and the other graded visually by experienced neuroradiologists. We analyzed both scores, which increases the validity of our results. Limitations include the fact that the qCS was assessed only on baseline CTA because no follow-up CTAs were available for review. Hence, we could not draw definitive conclusions about the evolvement of collateral status over time in individual patients, including any potential change between baseline imaging and the start of EVT, which was on average around 100 minutes. This delay can be attributed to the fact that around half of our patients presented in a primary stroke center and had to be transferred to an intervention center. We suggest this to be a focus of future studies, using sequential follow-up imaging, particularly in patients with persistent arterial occlusion. Furthermore, we used single-phase CTA, which may be inferior compared with multiphase CTA or timing-invariant CTA in the assessment of collateral status due to the influence of acquisition timing.^34,35^ However, we performed a sensitivity analysis excluding scans with suboptimal acquisition phases and found similar results. Furthermore, the use of single phase CTA improves applicability in clinical practice. Third, those with poor collateral status and presenting toward the end of the 6.5-hour time window may have been denied EVT and were therefore not included in the MR CLEAN Registry, which could have resulted in selection bias. However, guidelines do not recommend selection on the basis of collateral status in the first 6 hours, which makes major bias unlikely. Finally, because we excluded patients with an onset to groin puncture time of greater than 6.5 hours, the possibility exists that an interaction between time variables and collateral status may become apparent only after 6 hours from symptom onset.

In conclusion, this study shows that time to imaging is not associated with collateral status in patients with acute large vessel occlusion. Although this may suggest that flow into collaterals remains stable over time, studies using sequential neuroimaging assessments are needed to determine the course of collateral recruitment after large vessel occlusion over time with more certainty. Second, in the first 6.5 hours of symptom onset, collateral status does not modify the relation between time to recanalization and functional outcome, indicating that a shorter onset to recanalization time is equally beneficial across baseline collateral grades. Limiting time to recanalization should therefore remain a priority in all patients, regardless of collateral status.

## Glossary

acOR: adjusted common odds ratio
ASPECTS: Alberta Stroke Program Early CT Score
CTA: CT angiography
eTICI: extended Thrombolysis in Cerebral Infarction
EVT: endovascular treatment
HU: Hounsfield unit
ICA-T: internal carotid artery terminus
IQR: interquartile range
MR CLEAN: Multicenter Randomized Controlled Trial of Endovascular Treatment for Acute Ischemic Stroke
mRS: modified Rankin Scale
NIHSS: NIH Stroke Scale
qCS: quantitative collateral score
vCS: visual collateral score
